# Retraction Note: Graphene oxide (GO)-based nanosheets with combined chemo/photothermal/photodynamic therapy to overcome gastric cancer (GC) Paclitaxel resistance by reducing mitochondria-derived adenosine-triphosphate (ATP)

**DOI:** 10.1186/s12951-025-03805-0

**Published:** 2025-11-05

**Authors:** Weihong Guo, Zhian Chen, Xiaoli Feng, Guodong Shen, Huilin Huang, Yanrui Liang, Bingxia Zhao, Guoxin Li, Yanfeng Hu

**Affiliations:** 1 Department of General Surgery & Guangdong Provincial Key Laboratory of Precision Medicine for Gastrointestinal Tumor, Nanfang Hospital Southern Medical University, 510515 Guangzhou, China; 2 Guangdong Provincial Stomatology Hospital, Southern Medical University, Guangzhou, 510000 China; 3 Guangdong Provincial Key Laboratory of Cancer Immunotherapy, Guangzhou Key Laboratory of Tumor Immunology Research, Cancer Research Institute, School of Basic Medical Sciences, Southern Medical University, Guangzhou, 510515 PR China

**Retraction Note: Journal of Nanobiotechnology (2021) 19:146**

10.1186/s12951-021-00874-9

The Editor-in-Chief has retracted this article. After publication, a number of image integrity concerns were raised. Specifically:

In Fig. 2e, four panels were published in Fig. 1c in an article published previously sharing one of the same authors, showing different treatments [1].

In Fig. 7g, the three panels for PTX@GO-PEG-OSA-treated HGC-27/PTX cells, with addition of NAC, appear to overlap with the panels for PTX@GO-PEG-OSA-treated HGC-27/PTX cells, with exogenous ATP.

The authors attributed the apparent reuse of images to a shared platform used by both research groups, and were able to provide some original images but the nature of the images could not be verified by the publisher. The Editor has lost confidence in the data and conclusions of this article.

Author Yanfeng Hu disagrees with this retraction. All other authors did not respond to correspondence from the Publisher about this retraction.
